# Protective Effects of Salvianolic Acid A against Dextran Sodium Sulfate-Induced Acute Colitis in Rats

**DOI:** 10.3390/nu10060791

**Published:** 2018-06-19

**Authors:** Kai Wang, Qinqin Yang, Quanxin Ma, Bei Wang, Zhengrui Wan, Minli Chen, Liming Wu

**Affiliations:** 1 Institute of Apicultural Research, Chinese Academy of Agricultural Sciences, Beijing 100093, China; kaiwang628@gmail.com (K.W.); m13121180309@163.com (B.W.); 13515666376@163.com (Z.W.); 2 Zhejiang Institute of Traditional Chinese Medicine, Hangzhou 310007, China; qqy1029@126.com; 3 Comparative medical Research Center, Zhejiang Chinese Medical University, Hangzhou 310053, China; mqx1025@hotmail.com; 4 College of Bee Science, Fujian Agriculture and Forestry University, Fuzhou 350002, China

**Keywords:** *Salvia miltiorrhiza* Bunge, salvianolic acid A, inflammatory bowel disease, dextran sodium sulphate, gut microbiota

## Abstract

Salvianolic acid A (SAA) is an active phenolic acid derived from *Salvia miltiorrhiza* Bunge (Danshen). To explore whether SAA has a therapeutic effect against inflammatory bowel disease (IBD), an acute colitis model was induced in rats by administering 3% dextran sodium sulphate (DSS) for one week. SAA in doses of 4 and 8 mg/kg/day was given by tail vein injection during DSS administration. Both dosages of SAA ameliorated the colitis symptoms, with decreases observed in the disease activity index. A high dosage of SAA (8 mg/kg/day) promoted a longer colon length and an improved colonic tissue structure, compared with the DSS-treated rats not receiving SAA. SAA dose-dependently decreased colonic gene expression of pro-inflammatory cytokines (*IL-1β*, *MCP-1* and *IL-6*). Moreover, a high dosage of SAA protected against DSS-induced damage to tight junctions (TJ) in the rats’ colons, by increasing TJ-related gene expression (*ZO-1* and *occuldin*). Finally, using 16S rRNA phylogenetic sequencing, we found that SAA modulated gut microbiota imbalance during colitis by increasing the gut microbial diversity as well as selectively promoting some probiotic populations, including *Akkermansia* spp. Our study suggests that SAA is a promising candidate for the treatment of IBD.

## 1. Introduction

Inflammatory bowel disease (IBD), which includes Crohn’s disease (CD) and ulcerative colitis (UC), is a group of chronic disorders characterized by inflammation in the gastrointestinal tract [1]. The typical phenomena of UC occur in the inner lining of the rectum or the large intestine, causing frequent diarrhea, abdominal cramps, and rectal bleeding in some cases. In regard to Crohn’s disease, patients suffer from diarrhea, cramping abdominal pain, anorexia, weight loss, and occasional bleeding, due to damage generally found in deeper layers of the intestinal wall [2]. Even though the etiology of IBD remains uncertain, disruption of the intestinal mucosal immune system, defections of the intestinal mucosal barrier, and some genetic and environmental factors appear to be involved in the development of IBD [2]. Anti-inflammatory or immunosuppressive drugs, such as amino salicylic acid, steroid hormones, and 6-mercaptopurine, are the main drug currently used for IBD treatments. These drugs aim to induce remission and prevent relapse [3]. However, these treatments are associated with several limitations, including some side effects, drug intolerance, and a high recurrence rate. Therefore, there is a clinical need to identify novel and safe compounds for IBD prevention and treatment.

Recently, patients have turned to complementary and alternative medicine strategies, including traditional Chinese medicine (TCM), which include plant-based remedies [4]. A series of studies showed TCM-based formulas, plant extracts, or plant derivatives exert potent anti-inflammatory activity by controlling the release of various inflammatory cytokines, such as tumor necrosis factor α (TNF-α), interferon-γ (IFN-γ), interleukin (IL)-1, IL-6, and IL-12, as well as anti-inflammatory cytokines, including IL-4, IL-10, and transforming growth factor-β (TGF-β) [5]. Modern evidence for the efficacy of plant-based medicine against IBD has been systemically reviewed [6].

**Figure 1:**
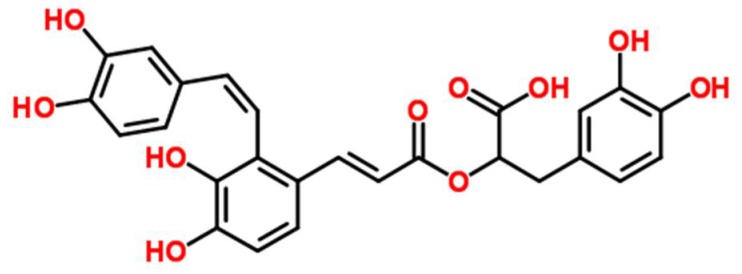
Chemical structure of salvianolic acid A (SAA).

*Salvia miltiorrhiza* Bunge (Danshen) is one of the most versatile oriental herbal plants used in TCM. It is a perennial which can be found throughout most parts of China and belongs to the Labiatae family [7]. Previous studies have shown its effectiveness against various inflammatory diseases, including atherosclerosis, arthritis, edema, and pancreatitis [8]. Researchers have also demonstrated its effectiveness within the gastrointestinal system, whereby it reduces inflammation in the small intestine and pancreas [9]. Recently, the effects of *Salvia miltiorrhiza* extract on chemically (azoxymethane and dextran sodium sulfate) induced colitis in mice was investigated, and the researchers found that it significantly reduced the disease activity index (DAI) as well as colon tissue lesions [10]. Salvianolic acid A (SAA), (2R)-3-(3,4-dihydroxyphenyl)-2-[(E)-3-[2-[(E)-2-(3,4-dihydroxyphenyl) ethenyl]-3,4-dihydroxyphenyl] prop-2-enoyl] oxypropanoic acid, (Figure 1) is a minor phenolic carboxylic acid from an aqueous extract of *S. miltiorrhiza*. A variety of pharmacological actions of SAA have been demonstrated, including antioxidant [11], cardioprotective [12], anti-inflammatory [13], antidiabetic [14], and antifibrotic effects [15]. A previous study has also shown that a water-soluble extract from *S. miltiorrhiza*, but not its constituent salvianolic acid B, inhibits endotoxin-induced nuclear factor (NF)-κB signaling in intestinal epithelial cells [16]. Nevertheless, the in vivo effects of SAA for gut health have not been extensively researched.

The objective of the present study was to assess whether salvianolic acid A can reduce the severity of colitis induced by dextran sodium sulphate (DSS) in rats. DSS-induced rodent colitis leads to a major shift in the population of the microbiota, which is similar to the changes that occur in human UC patients [17,18]. Therefore, we also explored whether the gut microbiota might play a role in any protection observed under SAA intervention.

## 2. Materials and Methods

### 2.1. Chemicals and Reagents

Salvianolic acid A (purity > 98%) was obtained from the Institute of Chiatai Qingchunbao Pharmaceutical Co., Ltd. (Hangzhou, China), DSS (M.W. 36–50 kDa) was purchased from MP Biomedicals (Irvine, CA, USA). All other reagents were obtained from Sangon Biotechnology (Shanghai, China) or as indicated in the specified methods.

### 2.2. Animals and Treatments

Male Sprague Dawley rats (six weeks old, ~185 g) were purchased from Shanghai Laboratory Animal Research Center (Shanghai, China). The study was a single experiment which was conducted in the Animal Experimental Center of the Zhejiang Institute of Traditional Chinese Medicine in Hangzhou, China. All animal experimental procedures were approved by the Institute of Apicultural Research of the Chinese Academy of Agricultural Sciences Animal Ethics Committee. The rats were housed in a room with controlled lighting (12 h light–dark cycle) and temperature (20 ± 2 °C). The laboratory diet was based on a standard AIN-93 laboratory diet (Xietong Biotechnology, Nanjing, China). The rats had free access to feed and water throughout the experimental study. They were acclimatized for four days before the study started, then randomly divided into four treatment groups of equal size (*n* = 8/group). The treatment groups were: (1) normal control, in which the rats received tap water and were not treated with DSS, (2) the DSS colitis control (received DSS), (3) salvianolic acid A low-dosage group, 4 mg/kg/day, i.v., and DSS), and (4) salvianolic acid A high-dosage group, 8 mg/kg/day, i.v., and DSS. SAA dosage was chosen on the basis of previously published literature [15]. Colitis was initiated by adding DSS (3%) to the drinking water daily for seven days (except for the normal control group), and all rats again received normal water for additional three days. SAA (10 mg/mL stock) was diluted in normal saline and given via daily tail vein injection, starting from the day of DSS administration until the end of the experimental study. The rats from the normal and DSS colitis controls were also injected intravenously with normal saline via the tail vein.

### 2.3. Disease Activity Index (DAI) Evaluations

Colitis was quantified using DAI evaluations based animal body weight, stools, and overall condition [18]. DAI was calculated daily from the start of DSS until the end of the experimental study. The scores were recorded based on previously published criteria, by summarizing the scores for body weight loss, stool consistency, rectal bleeding, and the overall animal condition. Each of these indices was scored on a 0–3 scale, with 0 representing no disease symptoms, and 3 representing severe disease symptoms.

### 2.4. Histologic Analysis

Distal colon samples were collected from the rats after culling, then washed in PBS and cut longitudinally. Half of the colonic tissue was subjected to histological analysis and fixed using 4% paraformaldehyde overnight. The remaining tissue was snapped frozen in liquid nitrogen and stored at −80 °C for subsequent measurement of mucosal mRNA expression. For hematoxylin and eosin (H&E) staining, distal colon tissue was dehydrated in a graded ethanol series, embedded in paraffin, and stained with hematoxylin and eosin. Colonic histological damage was observed under a light microscope.

### 2.5. Quantitative Real-Time Polymerase Chain Reaction (PCR) Analysis

Distal colon total RNA was extracted using an RNA Pure Kit (Carry Helix Biotechnologies Co., Ltd., Beijing, China). The reverse transcription was conducted using a PrimeScript RT Reagent Kit (TaKaRa, Dalian, China). Real-time PCR reactions were performed using SYBR Premix Ex Taq (TaKaRa) under the 7500c Real-time PCR Detection System (Applied Biosystems, Carlsbad, CA, USA). The primers were designed to flank introns with the Primer 5 software (Premier Biosoft, Palo Alto, CA, USA). The primer sets are listed in the Supplemental Table S1. The data were calculated using 2^−ΔΔ*CT*^ method, normalized to the expression of the housekeeping gene (*GAPDH*), and expressed as a fold change compared to the normal control group.

### 2.6. Microbiota Analysis

Gut microbiota was analyzed on the basis of our previous protocols [17]. In brief, total DNA was extracted from 150 mg of cecum digesta using the QIAamp DNA stool MiniKit (Qiagen, Hilden, Germany). PCR reactions targeting the V3–V4 regions of the 16S rDNA genes (probes used: 319F, 5′-ACTCCTACGGGAGGCAGCAG-3′ and 806R, 5′-GGACTACHVGGGTWTCTAAT-3′) were performed using the Premix Ex TaqTM Hot Start Version (Takara, Dalian, China), using a gradient PCR instrument (L96G; LongGene, Hangzhou, China). The sequencing reactions were subsequently performed using the Illumina Miseq^®^ sequencing technology (Illumina Inc., San Diego, CA, USA) for paired-end reads. The paired-end reads that were obtained were merged using FLASH v1.2.11 software, and reads with a length ≥400 pb were kept for the following analysis. The merged sequences were processed with QIIME v1.9.0, and the sequences were binned into operational taxonomic units (OTUs), based on 97% identity. Taxonomy analyses were performed using the Ramer–Douglas–Peucker (RDP) algorithm of the Greengenes database. Alpha diversity, richness (Chao1), Venn diagram, heat map, principal coordinate analysis, and linear discriminant analysis (LDA) effect size (LEfSe) were further processed with a bioinformatic pipeline tool, BMK Cloud online (http://www.biocloud.net/).

### 2.7. Statistical Analyses

Quantitative data are presented as the arithmetic mean ± standard deviation (SD) for each treatment group. The effect of the treatments was determined by one-way analysis of variance (ANOVA), and differences between treatments were analyzed post-hoc by Tukey’s honest significant difference test. A randomization/Monte Carlo permutation–based test (Adonis test) was chosen to determine significant differences between discrete and continuous variables. A p value less than 0.05 was considered statistically significant. All statistical tests were performed using SPSS version 17.0 (SPSS, Inc., Chicago, IL, USA).

## 3. Results

### 3.1. Effect of Salvianolic Acid A on DSS-Induced Colitis Symptoms

**Figure 2:**
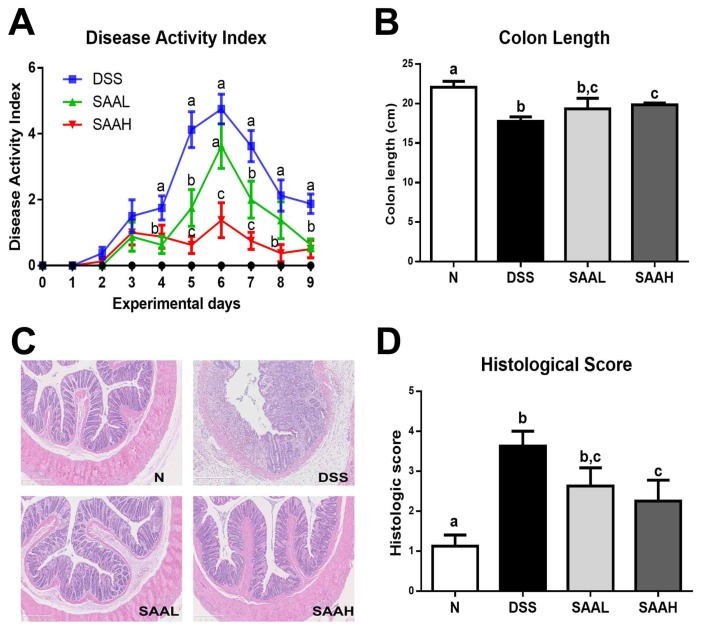
Salvianolic acid A ameliorated DSS-induced acute colitis symptoms in rats. The rats were administered DSS (4%) in drinking water for seven days. The SAA low-dosage group (SAAL, 4 mg/kg/day i.v.) and SAA high-dosage group (SAAH, 8 mg/kg/day i.v.) were tail vein-injected with SAA until the end of the experiment. (**A**) Disease activity index (DAI) values were recorded daily. (**B**) Colon length was recorded and compared among the normal control (N), DSS, SAA low-dosage group (SAAL), and SAA high-dosage group (SAAH). The data shown are the means ± SD, and means with different letters are significantly different (*p* < 0.05); *n* = 8 rats per group in a single experiment (**C**) Representative hematoxylin and eosin (H&E)-stained distal colon sections. (**D**) The histologic scores of rats. The data represent the mean ± SD of eight rats per group.

The DAI and colon length were measured, and histological examinations of the distal colon of the rats were performed to assess the protective effect of SAA. As shown in Figure 2A, DSS led to a significant increase in DAI values, peaking seven days after DSS administration, while the DAI of normal rats remained at zero throughout the whole experimental period. Importantly, these values were reduced in the SAA-injected groups (4 and 8 mg/kg body weight, b.w.). In addition, the weight gain in the SAA high-dosage group was significantly higher than in the DSS control group. No significant differences in final body weights were observed (Table 1). As shown in Figure 2B, significant colon shortening was noticed in the DSS group (17.8 ± 1.6 cm versus 22.0 ± 2.1 cm; *p* < 0.05), while SAA injections in the DSS-treated rats reduced this shortening of the colon. The histological examination of the distal colon of the rats showed serious microscopic mucosa inflammation, with typical observations including crypt damage, infiltration, ulceration, and edema in the intestinal epithelial layer in the DSS group, while SAA high-dosage group alleviated these pathological changes in the colon (Figure 2C,D) 

### 3.2. Salvianolic Acid A Decreased Inflammatory Gene Expression in the Colonic Mucosa of Rats after DSS-Induced Colitis 

**Figure 3:**
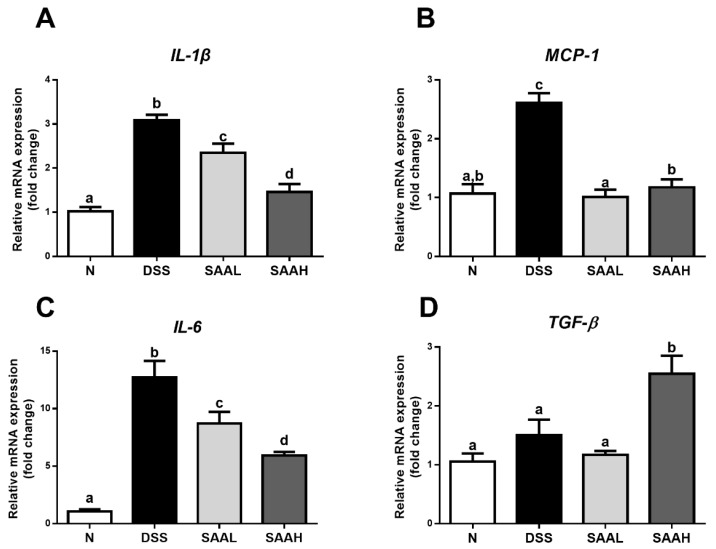
Effects of salvianolic acid A on inflammatory gene expression in the colonic mucosa of rats after DSS-induced colitis. The rats’ distal colons were collected, and mRNA expression of *TNF-α* (**A**), *IL-1β* (**B**), *IL-6* (**C**), and *TGF-β* (**D**) was quantified using real-time PCR. Fold changes are expressed as means ± SD (*n* = 8 for each group in a single experiment). Groups with different letters differ by a statistically significant margin (*p* < 0.05). N, normal control group; DSS, DSS colitis group; SAAL, SAA low-dosage group (SAAL, 4 mg/kg/day i.v.), and SAAH, SAA high-dosage group (SAAH, 8 mg/kg/day i.v.).

The effects of SAA on inflammatory gene expression in the colonic mucosa of rats after DSS-induced colitis is shown in Figure 3. Compared with the normal rats, significantly increased expression of pro-inflammatory cytokines (*IL-1β*, *MCP-1*, and *IL-6*) was noticed in the colonic mucosa tissues of DSS-treated rats. Moreover, injection with a high dosage of SAA rectified the over-expression of *IL-1β*, *MCP-1*, and *IL-6* (52.7 ± 11.8%, 55.0 ± 10.3%, and 53.4 ± 4.5%, respectively). The colonic expression of the anti-inflammatory cytokine *TGF-β* was significantly increased (1.69-fold) compared with the DSS group. Meanwhile, a low dosage of SAA also decreased *IL-1β*, *MCP-1*, and *IL-6* expression, but had no effect on *TGF-β* expression.

### 3.3. SAA Proteced against DSS-Induced Damage to Tight Junctions in the Colon of Rats

**Figure 4:**
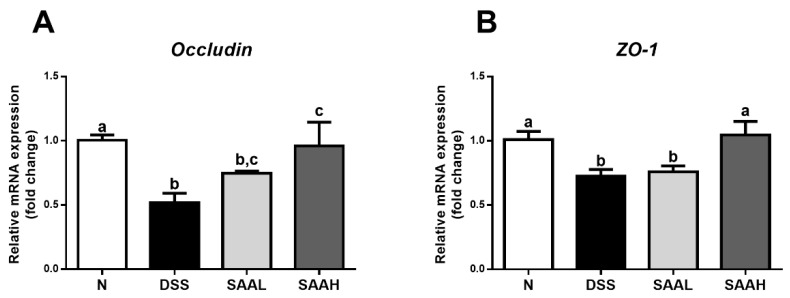
Effects of salvianolic acid A on the expression of genes coding for colonic tight junction proteins in DSS-induced-colitis rats. (**A**) The rats’ distal colons were collected, and mRNA expression of genes coding for the tight-junction proteins occludin (**A**) and ZO-1 (**B**) was quantified using real-time PCR. Fold changes are expressed as means ± SEM (*n* = 8 for each group in a single experiment). Groups with different letters differ by a statistically significant margin (*p* < 0.05).

The effects of SAA on the expression of genes coding for colonic tight junction proteins in DSS-induced-colitis rats is shown in Figure 4. The gene expression of colonic tight junctions was decreased dramatically in DSS-induced-colitis rats (*p* < 0.05). SAA injections increased the mRNA expression of *occludin* and *ZO-1*, compared with the DSS control.

### 3.4. Effects of SAA on Gut Microbial Composition in DSS-Induced-Colitis Rats

**Figure 5:**
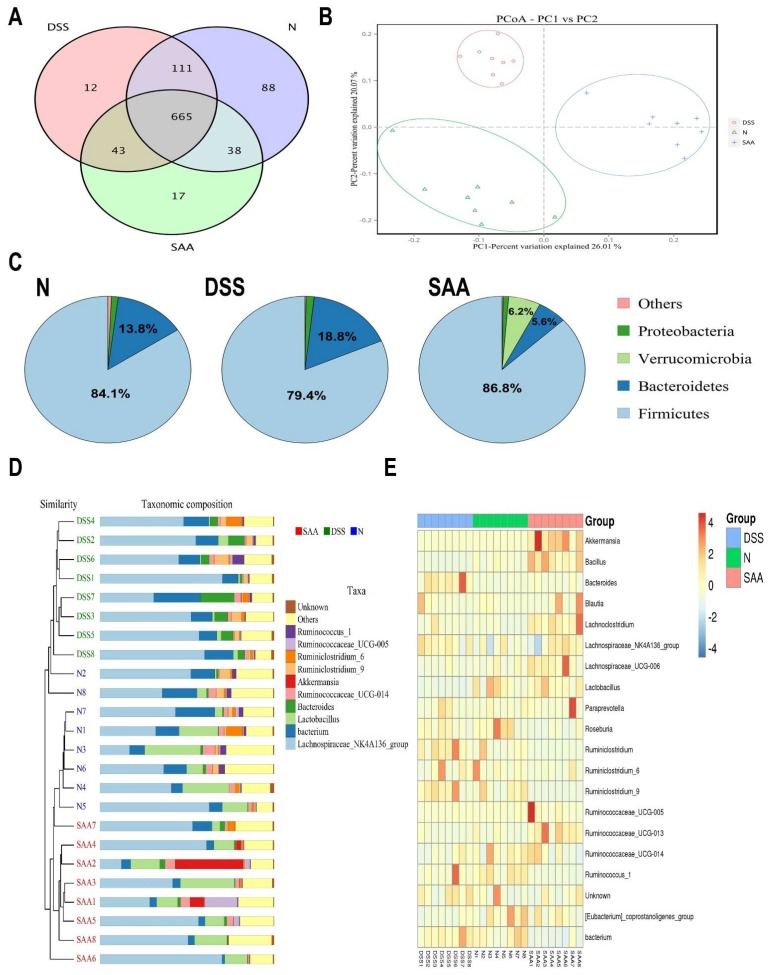
Effects of SAA on gut microbial composition in DSS-induced-colitis rats in a single experiment. (**A**) Venn diagram showing overlapping operational taxonomic units (OTUs) identified in the intestinal microbiota in the normal control, DSS, and SAA groups. (**B**) Principal coordinates analysis (PCoA) plot of the gut microbiota based on weighted UniFrac metrics. (**C**) Structural comparison of intestinal microbiota between the three groups at the phyla level. (**D**) Hcluster analysis of the gene levels for the three experimental groups. (**E**) Heat map of the 20 most differentially abundant taxons in the three groups at the gene level.

The cecal bacterial composition of the rats from the normal, DSS, and SAA high-dosage groups was measured by high-throughput pyrosequencing of the V3–V4 region of the 16S ribosomal DNA sequence. As shown in Table 2, no significant changes were observed between the average numbers of analyzed effective tags among these three groups. All the samples had Good’s coverages greater than 0.99. We further noticed that the alpha diversity of the microbial communities in the DSS group dropped significantly, with decreased microbial richness (indicated by ACE and Chao1 estimators) as well as less microbial diversity (indicated by an increased Simpson and dropped Shannon indexes). In addition, SAA increased the microbial alpha diversity to a level similar to that of the normal rats. The Venn diagrams showed overlapping OTU data for the three groups, which helped us understand the shared and distinct microbes. We found that 717 of 1054 OTUs were universal to all the groups. The SAA group had 17 distinct microbes, while distinct microbes in DSS and normal rats were 12 and 88, respectively (Figure 5A). The principal coordinates analysis (PCoA) graph clearly showed that the normal, DSS, and SAA groups have divided structures of gut microbiota, which were clustered into different quadrants (Figure 5B). The structures of the gut microbiome of the rats were significantly different from each other (Adonis test, *p* < 0.05). The analysis at the phylum level showed that Firmicutes and Bacteroidetes were predominant in the three groups, while their proportions were relatively different. DSS treatment increased the levels of Bacteroidetes by 36.2% and dropped the proportion of Firmicutes by 5.5%, compared with the normal control group. Following SAA injection, the Bacteroidete and Firmicute proportions returned to normal control levels. Moreover, SAA increased the microbial proportion of the phyla *Verrucomicrobia*, which is different to what was observed in the other two groups (Figure 5C). At the gene level, the hcluster analysis results showed that the gut microbiota in the rats of these three groups were evolutionarily different compared to that of other groups (Figure 5D). As shown in Figure 5E, we generated a heat map showing 20 genera with a high frequency and their relative abundance. *Akkermansia* spp., *Bacillus* spp., *Blautia* spp., *Lachnoclostridium* spp., and *Lactobacillus* spp. were increased following SAA injection, and this was accompanied by a decrease in the relative abundance of the *Bacteroides* spp., *Roseburia* spp., and *Ruminiclostridium* spp.

## 4. Discussion

Salvianolic acid A is known as one of the major bioactive compounds in *S. miltiorrhiza* Bunge (Danshen), which has been used for years in traditional Chinese medicine for the treatment of cardiovascular diseases. On the basis of TCM concepts, *S. miltiorrhiza* is commonly used for cardiovascular illness treatment because of its effectiveness in alleviating blood stasis, exciting blood circulation, and nourishing the cardiovascular system. Recently, a number of studies have revealed that it has excellent anti-inflammatory effects, including in gastrointestinal inflammation. Wen et al. noticed that an aqueous extract of *S. miltiorrhiza* showed promising anti-colitis effects in a DSS-induced-colitis mice model [10]. As SAA is the main active component of the water-soluble phenolic acid derivatives of *S. miltiorrhiza*, we designed a study to investigate the effects of SAA in DSS-induced acute colitis. Apart from the anti-inflammatory effects demonstrated by SAA in the present study, we also noticed its positive effects in improving the intestinal epithelial barrier and in modulating the gut microbiota during colitis.

Some macroscopic indices measured in the animal experimental colitis model reveal colitis severity, including DAI, colon length, and body weight loss. The histological examination of colon sections shows intestinal pathological changes [19]. In parallel with a previous study using a water-soluble extract of *S. miltiorrhiza*, we also found that SAA showed a healing effect against DSS-induced rodent colitis, by dropping the DAI, increasing the shortened colon length, and reversing the decreased body weight [10]. Histological characterizations also confirmed that intestinal inflammation was alleviated by SAA injections.

A variety of cytokines participate in the pathogenesis and therapeutic mechanisms of IBD [20,21]. These cytokines are also involved in the regulation of intestinal mucosal inflammation and intestinal epithelium integrity [22]. Clinical samples from IBD patients showed that some pro-inflammatory cytokines, like TNF-α, are highly correlated with colitis disease activity [22]. Hence, recent studies have noticed that the modulation of inflammatory cytokines during IBD has great therapeutic application potential. In the present study, we found that SAA-administrated colitis rats showed decreased expression of the pro-inflammatory cytokines IL-1β, IL-6, and MCP-1. Indeed, previous research has reported that SAA has good anti-inflammatory effects due to the inhibition of inflammatory mediators’ release in both in vitro and in vivo studies [13]. In macrophages, SAA significantly suppresses TNF-α–induced CC chemokine ligand-20 (CCL-20) release [23]. Similar results were also observed in ApoE-deficient (ApoE−/−) mice [24]. Pretreatment with SAA suppressed the endotoxin-induced production of nitric oxide and prostaglandin E2 in murine RAW 264.7 macrophages [13]. It has been reported that TGF-β is an anti-inflammatory cytokine during the recovery of inflammation-related colitis [25]. However, in this research, we found that SAA increased TGF-β expression in the colon, which is inconsistent with the results of a previous study. In that particular study, SAA was found to alleviate the lesions of hepatic steatosis and fibrosis in a streptozotocin-induced diabetes model administered a high-fat diet, via inhibition of α-smooth-muscle-actin (α-SMA) and TGF-β in the liver [26]. Therefore, we hypothesize that the anti-colitis effect of SAA might be attributed to its regulation of inflammatory mediators.

An intact intestinal barrier is extremely important for maintaining a healthy gut and preventing colonization by pathogens. DSS-induced acute colitis is known to dramatically increase intestinal permeability, which is the result of broad intestinal epithelial cell damage as well as loss of gut tight junction barrier functions. Since tight junction protein expression and distribution patterns are the main determining factors for gut barrier function, the upregulation by SAA of the expression of genes coding for tight junction proteins (ZO-1, occludin) provides another explanation for SAA anti-colitis effects. A recent study reported that the aerial parts of *S. miltiorrhiza* boosted tight junction protein expression (ZO-1, occludin, and claudin-5) in the ileum and colon in streptozocin-induced diabetic mice, providing the necessary evidence for alternative therapies for diabetic enteropathy [27]. As SAA was also identified in the aerial parts of *S. miltiorrhiza*, we inferred that the gastric protective mechanisms of SAA were due, at least in part, to its modulating effect on gut barrier function.

The gut microbial populations play important roles in maintaining physiological functions in the host. More recently, studies have been focused on the potential interactions between the gut microbiota and the active components of herbal medicine [28,29]. A variety of herbal medicines (including individual active compounds, herbal extracts, and medicinal formulas) have shown the capability to reverse or mitigate gut microbial dysbiosis associated with diseases like cancer, neurodegenerative diseases, chronic kidney disease, metabolic diseases, and IBD [30,31]. Although previous studies have shown the potential of *S. miltiorrhiza* extracts to modulate gut microbial populations [27], the evidence for its bioactive constituents remains limited. Here, using high-throughput pyrosequencing analysis, we explored SAA modulating effects on the gut microbiota in DSS-induced colitis. Patients with IBD have been occasionally found to have microbial dysbiosis with an increased Bacteroidetes/Firmicutes ratio. Compared with the DSS control, we noticed that this ratio was decreased by SAA injections, suggesting that SAA has promising effects in maintaining gut homeostasis. Recent studies suggest that *Akkermansia*, a Gram-negative anaerobe belonging to the *Verrucomicrobia*, is an emerging probiotic in the gut [32]. In IBD patients (mainly UC patients), reduced levels of *Akkermansia* have been observed [33]. Studies have also demonstrated that *Akkermansia* protects against DSS colitis [34,35]. We found that the *Verrucomicrobia* phyla was increased by SAA, and *Akkermansia* spp. also became the predominant bacteria genera in the gut [36]. Therefore, we infer that SAA led to changes in the gut microbiota composition and selectively increased some probiotics, like *Akkermansia* spp. Nevertheless, whether microbial dysbiosis in the gut is a cause or an outcome of gut inflammation in colitis rats remains controversial. We inferred that SAA inhibits inflammation and thereby prevents the microbiota shift caused by the inflammatory responses. It should be noticed that the current study was focused on the assessment of the therapeutic effects of SAA against acute colitis in a single experiment, and we did not provide adequate information on its reproducibility. Moreover, despite the fact that the anti-colitis effect of SAA was shown to be dose-dependent, the modulating effects by different dosages on the gut microbial profiles still need to be explored in the future.

Taken together, we have shown here that tail vein injections of salvianolic acid A, which is the major active compound present in *S. miltiorrhiza* Bunge, significantly alleviated the acute colitis symptoms induced by DSS in rats. In this single experiment, SAA injections not only relieved macroscopic pathological manifestations, such as the disease activity index, but also alleviated intestinal inflammation and modulated DSS-induced barrier leakiness. We also showed that SAA intervention may be beneficial for the stability of the gut microbiota, by promoting the growth of probiotic bacteria selectively, including *Akkermansia* spp.

## Figures and Tables

**Table 1:** Body weight, organ index, and fad pad weights of the rats ^1^.

Parameters	Normal	DSS	SAA (4 mg/kg)	SAA (8 mg/kg)
Body weight (g)	321.14 ± 9.06 ^a^	298.63 ± 12.13 ^b^	305.21 ± 17.1 ^a,b^	315.9 ± 12.02 ^a^
Liver weight (mg/g)	40.67 ± 4.62 ^a^	43.58 ± 6.93 ^b^	39.66 ± 2.77 ^a^	36.66 ± 3.27 ^c^
Spleen weight (mg/g)	2.32 ± 0.44	2.6 ± 0.51	2.93 ± 0.65	2.94 ± 0.41
Kidney weight (mg/g)	8.09 ± 0.71	8.68 ± 0.59	7.95 ± 1.71	8.08 ± 0.33
Epididymal adipose tissue (mg/g)	7.2 ± 1.64	7.45 ± 1.62	6.84 ± 3.23	8.38 ± 2.17
Retroperitoneal adipose tissue (mg/g)	6.29 ± 2.22	7.49 ± 2.52	7.1 ± 2.06	7.39 ± 2.35

^1^ Data are expressed as means ± SD (*n* = 8 in a single experiment). Means with different letters are significantly different (*p* < 0.05).

**Table 2:** Diversity indices obtained from the pyrosequencing results ^1^.

Groups	Effective Tags			Good’s Coverage			ACE Estimators			Chao1 Estimators			Simpson Indices			Shannon Indices		
N	38,053	±	3143	0.996	±	0.001	675	±	20a	691	±	18a	0.0608	±	0.0070 ^a^	4.1	±	0.1 ^a^
DSS	36,479	±	2008	0.996	±	0.001	521	±	21b	537	±	19c	0.1526	±	0.0215 ^b^	3.3	±	0.1 ^b^
SAA (8 mg/kg)	32,925	±	1770	0.997	±	0.001	629	±	10a	639	±	13b	0.0471	±	0.0052 ^a^	4.2	±	0.1 ^a^

^1^ Data indicates means ± SEM (*n* = 8 in a single experiment). Groups with different letters are statistically different (*p* < 0.05).

## Supplementary Materials

The following are available online at http://www.mdpi.com/2072-6643/10/6/791/s1, Table S1: Primer Sequences used for qRT-PCR.
