# Supplementary material for: Protective Effects of Salvianolic Acid A against Dextran Sodium Sulfate-Induced Acute Colitis in Rats
Source: Nutrients. 2018 Jun 19;10(6):791. doi: 10.3390/nu10060791 (PMC6024375; doi:10.3390/nu10060791)
Supplement: Supplementary file 1 [file nutrients-10-00791-s001.pdf]

Supplemental Table 1. Primer Sequences used for qRT-PCR

Gene		Primer sequence	Product size (bp)	GenBank accession No.
GAPDH	F:	CAAGTTCAACGGCACAGTCA	100	NM_017008.4
	R:	CCATTTGATGTTAGCGGGAT		
IL-1β	F:	CTCGTGCTGTCTGACCCAT	247	NM_031512.2
	R:	CAAACCGCTTTTCCATCTTC		
MCP-1	F:	AGCAGGTGTCCCAAAGAAGC	251	NM_031530.1
	R:	ACAGAAGTGCTTGAGGTGGT		
IL-6	F:	TGGAGTTCCGTTTCTACCTG	220	NM_012589
	R:	TGGATGGTCTTGGTCCTTAG		
TGF-β	F:	GGCGGTGCTCGCTTTGTA	135	NM_021578.2
	R:	ATTGCGTTGTTGCGGTCC		
Occludin	F:	AGGCTTCTGGATCTATGTACG	208	NM_031329.2
	R:	ATCTTTCTTCGGGTTTTCAC		
ZO-1	F:	CAGCCCCAGGTAGTGAGT	163	NM_0011062 66.1
	R:	GAGGACCGTGTAATAGCAGA		
